# Osteopontin Bridging Innate and Adaptive Immunity in Autoimmune Diseases

**DOI:** 10.1155/2016/7675437

**Published:** 2016-12-20

**Authors:** Nausicaa Clemente, Davide Raineri, Giuseppe Cappellano, Elena Boggio, Francesco Favero, Maria Felicia Soluri, Chiara Dianzani, Cristoforo Comi, Umberto Dianzani, Annalisa Chiocchetti

**Affiliations:** ^1^Department of Health Sciences and Interdisciplinary Research Center of Autoimmune Diseases (IRCAD), “A. Avogadro” University of Piemonte Orientale (UPO), Novara, Italy; ^2^Biocenter, Division for Experimental Pathophysiology and Immunology, Laboratory of Autoimmunity, Medical University of Innsbruck, Innsbruck, Austria; ^3^Department of Drug Science and Technology, University of Torino, Torino, Italy; ^4^Department of Translational Medicine, Neurology Unit, “A. Avogadro” UPO, Novara, Italy

## Abstract

Osteopontin (OPN) regulates the immune response at multiple levels. Physiologically, it regulates the host response to infections by driving T helper (Th) polarization and acting on both innate and adaptive immunity; pathologically, it contributes to the development of immune-mediated and inflammatory diseases. In some cases, the mechanisms of these effects have been described, but many aspects of the OPN function remain elusive. This is in part ascribable to the fact that OPN is a complex molecule with several posttranslational modifications and it may act as either an immobilized protein of the extracellular matrix or a soluble cytokine or an intracytoplasmic molecule by binding to a wide variety of molecules including crystals of calcium phosphate, several cell surface receptors, and intracytoplasmic molecules. This review describes the OPN structure, isoforms, and functions and its role in regulating the crosstalk between innate and adaptive immunity in autoimmune diseases.

## 1. Introduction

OPN is an acidic glycoprotein that, depending on its intracellular (iOPN) or extracellular (OPN) localization, is involved in inflammation by inducing cell adhesion and migration, regulating the differentiation of proinflammatory lymphocytes, and inhibiting the apoptosis of inflammatory cells. It was initially described as a bone-specific sialoprotein [1] and then as a molecule expressed in activated T cells, consequently being named the “early T cell activated gene” (ETA-1) [2, 3].

OPN is produced by a variety of cell types, such as B and T cells, natural killer (NK) cells, NKT cells, macrophages, neutrophils, dendritic cells (DC), bone cells (osteoblasts and osteocytes), breast epithelial cells, and neurons, and high expression is detected in the bone, lung, liver, brain, joints, adipose tissue, and body fluids such as blood, urine, and milk [4–6].

### 1.1. *OPN* Gene

**Figure 1:**
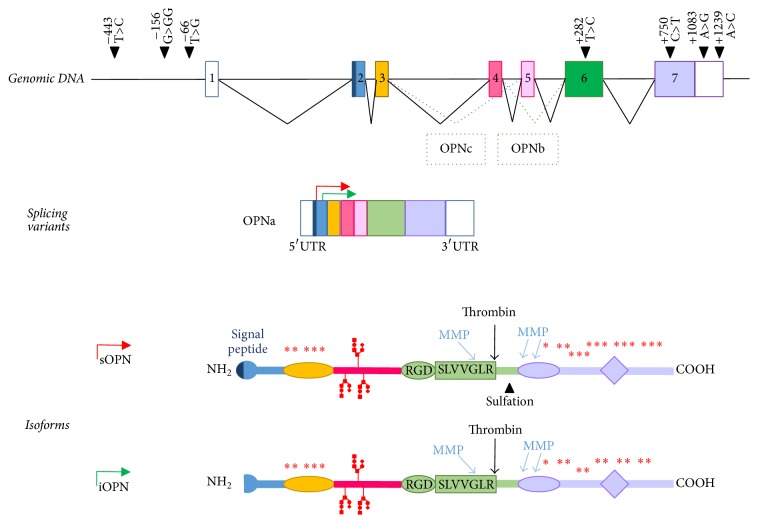
Genomic, transcriptional, and protein features of OPN. The figure shows in the upper panel the genomic organization of the* SPP1* gene and the relevant single nucleotide polymorphisms. OPN is transcribed with 3 splicing variants: variant a contains exons 2–7 while variants b and c lack exons 5 and 4, respectively (middle panel). OPN transcripts have also two starting points generating a secreted or/and intracellular form. Several posttranslational modifications are also shown (lower panel) including phosphorylation (asterisks), glycosylation, and sulfation sites. Proteases (thrombin and matrix metalloproteinase, MMP) cleavage sites are also depicted.

OPN is encoded by an 8 kb gene mapping on chromosome 4q13 and composed of 7 exons; the first exon is untranslated while exons 2–7 contain the coding sequences (Figure 1). Genetic variations of the* OPN* gene have been described in the 5′ flanking region, exons, introns and the 3′ untranslated region (3′UTR) [7–9]. Some of these variations are associated with development and/or disease activity of several autoimmune diseases [10–13] and some of them influence OPN expression [14]. For instance, the four single nucleotide polymorphisms (SNPs) +282T>C (exon VI: rs4754), +750C>T (exon VII; rs1126616), +1083A>G (3′UTR; rs1126772), and +1239A>C (3′UTR; rs9138) are associated with three haplotypic combinations, that is, 282T-750C-1083A-1239A (haplotype A), 282C-750T-1083A-1239C (haplotype B), and 282C-750T-1083G-1239C (haplotype C), and carriers of haplotype B and haplotype C display higher OPN serum levels and higher risk of developing several autoimmune diseases than haplotype A homozygotes. This effect may be related to the higher stability of the mRNA coded by haplotype B and haplotype C compared to that coded by haplotype A [15]. Interindividual differences of OPN expression may be also influenced by variations in the promoter region, such as −66T>G (rs28357094) and −156G>GG (rs7687316) SNPs, which may modulate the transcriptional activity of the gene [15–18].  Table 1 summarizes several associations reported between OPN SNPs and autoimmune diseases (http://www.ncbi.nlm.nih.gov/projects/SNP/) [10, 13–16, 19–25].

### 1.2. OPN Structure

The protein is composed of 314 amino acids, rich in aspartate, glutamate, and serine residues, and it contains functional domains for calcium binding [26]. Its molecular weight ranges from 44 to 75 kDa, depending on alternative splicing and posttranslation modifications. Given its composition, OPN is a highly negatively charged protein lacking extensive secondary structure that displays 8 *α*-helices and 6 *β*-sheets. Besides the full-length variant, named OPN-a and containing all exons, two splicing variants are OPN-b and OPN-c lacking exon 5 and exon 4, respectively [27]. All of them use exon 2, which contains the signal peptide, and are expected to be secreted. Another form derives from alternative initiation of transcription, lacks the signal peptide, and is expressed as an intracellular protein (iOPN) (Figure 1). As both OPN and iOPN are generated from the same mRNA, selective silencing of only one of these isoforms is not possible. OPN deficient mice lack both forms, while administration of OPN neutralizing antibodies or aptamers selectively blocks only OPN [28]. Other variants depend on several posttranslational modifications, including phosphorylation, O-linked glycosylation, sialylation, and tyrosine sulfation [29–34].

### 1.3. OPN Functions

OPN functions as a free cytokine in body fluids or as an immobilized extracellular matrix molecule in mineralized tissue. Its pleiotropic effects are partly due to its capacity to interact with multiple ligands including several cell surface receptors, intracellular signaling molecules, calcium, and heparin.

The binding sites to cell surface receptors include a RGD (arginine–glycine–aspartate) motif interacting with integrins *α*v*β*1, *α*v*β*3, *α*v*β*5, *α*v*β*6, *α*8*β*1, and *α*5*β*1 [35–37], and a binding site for CD44, in particular for the isoform CD44v_6_-v_7_. Moreover, thrombin cleaves OPN at a conserved site (^168^RS^169^) and exposes a cryptic ^162^SVVYGLR^168^ motif interacting with the integrins *α*9*β*1, *α*4*β*1, and *α*4*β*7 [38, 39]. The RGD and the cryptic sites are located in the N-terminal fragment of OPN produced by thrombin cleavage (OPN-N), whereas the CD44 binding site is located in the corresponding C-terminal fragment (OPN-C).

**Figure 2:**
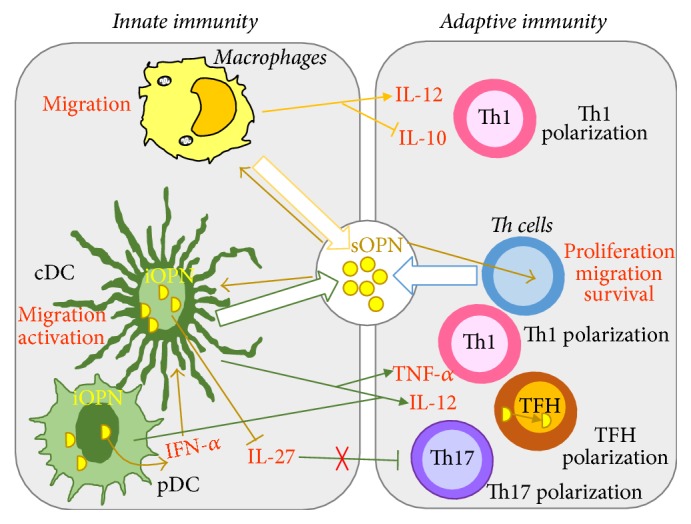
OPN mediates innate-adaptive immune crosstalk. Soluble OPN (OPN) acts on macrophages upregulating interleukin- (IL-) 12 production and mediates T helper (Th) 1 development. It also acts on Th cells, inducing the production of IL-17 and inhibiting secretion of IL-10 resulting in Th17 polarization. In conventional dendritic cell (cDC), iOPN inhibits IL-27 expression and enhances the response of Th17 cells. In plasmacytoid DC (pDC), it enhances interferon (IFN) *α* expression. iOPN has also a key role in T follicular helper (TFH) cells, since during activation iOPN translocate into the nucleus and sustains TFH polarization. Big and empty arrows show OPN production; thin arrows indicate OPN actions.

The role of OPN in the crosstalk between innate and adaptive immunity (iOPN is described later) is clearly highlighted in the development of proinflammatory T helper (Th) type-1 and Th17 cells (Figure 2). By acting on macrophages, OPN upregulates interleukin- (IL-) 12 production and enhances Th1 development. By acting on Th cells, OPN induces production of IL-17 by triggering *α*v*β*3 integrin and inhibits secretion of IL-10 by triggering CD44 [40]. By interacting with CD44 in Th cells, OPN induces hypomethylation of interferon- (*IFN-*)*γ* and* IL-17a* genes enhancing differentiation of Th1 and Th17 cells. In contrast, CD44 deficiency promotes hypermethylation of* IFN-γ* and* IL-17a* and hypomethylation of* IL-4* gene, leading to Th2 cell differentiation [41].

We have recently demonstrated that OPN-N and OPN-C generated by thrombin-mediated cleavage display distinct functions. In T cells, OPN-N promotes IL-17 secretion, whereas OPN-C inhibits IL- 10 secretion. In monocytes, secretion of IL-6 is induced mainly by OPN-N. In several cell types, including vascular endothelial cells and tumor cells, OPN-N induces migration whereas OPN-C induces adhesion. By contrast, both fragments similarly induce IFN-*γ* secretion in T cells and tubulogenesis in vascular endothelial cells and inhibit activation-induced cell death in lymphocytes [42].

Human OPN has two strong heparin-binding domains associated with internalization signals, which suggests that it rapidly binds to surface heparan sulfate proteoglycans to be internalized. Interestingly, the thrombin cleavage site is located close to one of these heparin-binding domain. Thus, heparin binding to OPN blocks the access of thrombin and maintains OPN in the full-length form. Furthermore, the N-terminal of OPN is dominated by acidic, negatively charged amino acids, whereas all of the positively charged heparin-binding domains are on the C-terminal part [43, 44].

The biological activity of OPN can be modulated also by cleavage mediated by several matrix metalloproteinases (MMPs), including MMP-1, MMP-2, MMP-3, MMP-8, MMP-9, MMP-10, MMP-11, MMP-12, MMP-13, MMP-14, and MMP-25. These are increased in biological samples of patients with autoimmune diseases, and the majority of them have a detrimental role since their activity inhibit cell adhesion and/or the migration driven by OPN [45, 46]. Human OPN contains three cleavage sites for MMPs located between the amino acids Gly^166^-Leu^167^, Ala^201^-Tyr^202^, and Asp^210^-Leu^211^. Interestingly, MMP-12 processes OPN into a less-inflammatory form, or, alternatively, it generates OPN peptides with anti-inflammatory properties [47].

Other mechanisms controlling OPN functions are posttranslational modifications, including O-linked glycosylation, sialylation, phosphorylation, and tyrosine sulfation, which influence each other and complicate the functional study on OPN and its variants [48]. For instance, O-GlcNAcylation antagonizes phosphorylation in terms of abundance, protein distribution, and activity of the protein [49]. Moreover, reduction of sialylation may prevent OPN from binding to cell surface receptors [31].

OPN displays substantial O-glycosylations, while the level of N-glycosylation seems to be low. Accurate analysis of O-glycosylation on human OPN detected multiple complex and heterogeneous glycans and *α* (2-3) sialic acids and a novel O-glycosylation site (S146) in the SVVYGLR domain which might regulate the interaction of OPN with integrins [50, 51]. Additionally, steric hindrance of glycan structures on S146 may impair the OPN cleavage mediated by MMPs. Other O-glycosylation sites are located in the C-terminus of OPN, which binds to CD44 and may regulate various cellular events, including binding interactions [52].

OPN displays 26 phosphorylation sites which may interplay with the O-glycosylation sites [53]. Nevertheless, one new phosphorylation site, Y209, was detected. Interestingly, among the identified O-glycopeptides, aa205–225, aa234–252, and aa286–298 were also identified as phosphorylation sites, indicating that interplay between O-glycosylation and phosphorylation on these sites may occur in OPN.

OPN phosphorylation seems to play regulatory roles in bone mineralization, OPN-receptor interactions, and tumor metastasis [54].

OPN regulates bone mineralization and has diverse effects on hydroxyapatite (HA) formation and growth depending on the extent of phosphorylation, since phosphorylated OPN can bind to Ca^2+^ and Mg^2+^ which are essential bone components. Phosphorylated OPN from bone inhibits hydroxyapatite mineral deposition in both cell-free systems [55–58] and in cell cultures [59–62]. In contrast the highly phosphorylated milk OPN promotes mineralization in solution [63]. Moreover, in OPN-KO mice, mineralization is enhanced in bone, calcified cartilage [64], vasculature [65], and kidney [66]. Bovine OPN has two thrombin cleavage sites, thus generating three thrombin-cleaved fragments: an N-terminal, a central, and a C-terminal fragment. These fragments have distinct effects on HA formation and growth: the central fragment is an inhibitor of HA formation, while the C- and N-terminal fragments promote HA formation [67].

Sulphate groups of OPN have been shown to act cooperatively with polyaspartic acid peptides in a *β*-sheet structure in promoting HA formation. Accordingly, Nagata et al. showed that sulfation of OPN is important for early formation of HA crystals in bone, is a valuable indicator of bone formation, and marks the osteoblastic phenotype [68].

### 1.4. iOPN

iOPN was initially found in rat calvarial cells [69] showing two patterns of OPN staining: a perinuclear staining located in the Golgi apparatus and a perimembrane staining reminiscent of focal adhesion staining [69]. In addition, staining was also detected in the nucleus [70]. It was initially suggested that iOPN plays a role mainly in innate immunity, since DCs and macrophages constitutively express high levels of OPN mRNA but secrete relatively small amounts of OPN. By contrast, activated T cells produce 50-fold more OPN than macrophages [71, 72]. The function of iOPN seems to be mainly involved in supporting signaling through several receptors. In plasmacytoid DC (pDC), it is involved in toll-like receptor- (TLR-) 9/TLR-7 by “anchoring” multiple pattern-recognition receptors (PRRs) to form receptor clusters. Moreover, TLR9 ligation promotes association of iOPN and myeloid differentiation primary response gene 88 (MyD88) and enhances IFN-*α* expression through interferon regulatory factor (IRF) 7 activation. In conventional DC, iOPN inhibits IL-27 expression and enhances the response of Th17. In macrophages, it promotes nuclear translocation of interleukin-1 receptor-associated kinase 1 (IRAK1) and IL-10 expression. Recent data have detected a key role of iOPN in T follicular helper (TFH) cells, since inducible T cell costimulator (ICOS) signaling induces iOPN interaction with the PI3K p85*α* regulatory subunit, followed by translocation into the nucleus and binding to B cell lymphoma- (Bcl-) 6 (involved in TFH differentiation) protecting it from proteasome-mediated degradation [73]. Moreover, at perimembrane regions, iOPN colocalizes with CD44-ERM- (ezrin-radixin-moesin-) actin complexes, and it is involved in cell motility [74]; this activity seems to play a key role in the osteoclast function [75, 76].

## 2. OPN and Autoimmune Diseases

In the past thirty years, OPN has attracted attention following observations that high levels of OPN can be detected in several autoimmune diseases, like systemic lupus erythematosus (SLE) [77, 78], multiple sclerosis (MS) [79], rheumatoid arthritis (RA) [80], and others [14, 81]. In line with these observations, transgenic overexpression of OPN in a nonautoimmune background causes accumulation of B cells, hypergammaglobulinemia, and production of anti-DNA antibodies, which is typical of SLE [82]. Moreover, OPN deficient mice are relatively protected against MS [83], RA [84], type 1 diabetes mellitus (T1DM) [85], autoimmune uveitis [86], autoimmune hepatitis [87, 88], intestinal bowel disease (IBD) [89], and Sjögren's syndrome (SS) [90, 91].

The detrimental activity of OPN in autoimmune diseases may involve its ability to promote secretion of IL-17 and IFN-*γ* in T cells and IL-6 in monocytes, to promote lymphocyte adhesion and migration, to inhibit activation-induced cell death that is involved in the switching off the immune response and to support TFH differentiation (Figure 2).

Genetic investigations, including genome-wide association studies (GWAS), have identified numerous, replicable, genetic associations between common SNPs and susceptibility to autoimmune disease, some of which are shared between two or more diseases [92, 93]. Along with epidemiological and clinical evidence, this suggests that some genetic risk factors may be clustered into groups and influence entire pathways to create risk to multiple diseases [94]. Unexpectedly, no GWAS identified OPN SNPs as associated to any autoimmune disease. Nevertheless, GWAS approach has been enriched and complemented by genome-wide expression profiling, in ex vivo innate (NK and monocytes) or adaptive immune cells (CD4+ T cells, B cells) [92]. Interestingly, genome-wide differential analysis in SLE identified distinct inflammatory pathways involved, including OPN [95].

In this part of the manuscript, we will review the data on SLE, MS, and RA, which display a huge literature related to OPN. They are also mediated by distinct immunopathologic features, since SLE is mainly mediated by antibodies and MS by T cells, whereas RA damage involves bone erosions. Recent advances in other autoimmune diseases are also discussed at the end.

### 2.1. Systemic Lupus Erythematosus

SLE is a complex autoimmune disease characterized by production of autoantibodies (autoAbs) against nuclear, cytoplasmic, and cell surface molecules that transcend organ-specific boundaries. Tissue deposition of antibodies or immune complexes induces inflammation and subsequent injury of multiple organs and finally results in clinical manifestations of SLE, including glomerulonephritis, dermatitis, thrombosis, vasculitis, and arthritis [96, 97].

The first evidence of a relationship between OPN and SLE was reported in MRL^*lpr*/*lpr*^ mice, developing a disease partly resembling SLE [98]. MRL^*lpr*/*lpr*^ mice were found to carry a loss-of-function mutation of the* FAS* gene, which was followed by identification of the corresponding human disease, which was named autoimmune lymphoproliferative syndrome (ALPS). MRL^*lpr*/*lpr*^ mice and ALPS patients are characterized by accumulation of polyclonal lymphocytes in the secondary lymphoid organs, expansion of a peculiar subset of T cells expressing TCR*αβ* but not CD4 or CD8 (named double negative T cells) and autoimmune manifestations. MRL^*lpr*/*lpr*^ mice, but not ALPS patients, produce anti-DNA autoAbs and develop vasculitis, arthritis, and glomerulonephritis causing fatal renal failure [99–101]. In both MRL^*lpr*/*lpr*^ mice and ALPS patients, the disease is due to decreased function of the proapoptotic FAS receptor involved in switching off the immune response. Most ALPS patients carry an inherited or somatic loss-of-function mutation of* FAS*. Rare ALPS patients carry mutations of* FASLG* coding for FAS-ligand, or* CASP10* coding for caspase 10 involved in FAS signaling; a mutation of* FASLG* is carried also by MRL^*gld*/*gld*^ mice showing a disease similar to that displayed by MRL^*lpr*/*lpr*^ mice. However, a substantial proportion of ALPS patients display a defective function of FAS in the absence of known mutations, which is a pattern shown also by patients with Dianzani autoimmune lymphoproliferative disease (DALD) displaying lymphoproliferation and autoimmune manifestations but lacking DN T cell expansion [102, 103].

In mice, CD4^−^ CD8^−^ T cells expressed high levels of OPN. Overexpression of OPN in MRL^*lpr*/*lpr*^ mice induces B cell activation and IgG and IgM production, elevated autoantibodies levels (including autoAbs to double-stranded (ds) DNA), and increased cytokine expression (TNF-*α*, IFN-*γ*, and IL-1*β*) [12, 82, 97]. In MRL^*lpr*/*lpr*^ mice, OPN upregulation begins at the onset of the autoimmune response and positively correlates with the symptom severity [86], which is also influenced by allelic differences in* OPN* gene [12, 104], since development of glomerulonephritis is favored by the* OPN* variant carried by MRL strain but not by the one carried by the C3H strain. These allelic variants correlate with different levels of antibody production, tumor necrosis factor- (TNF-) *α*, IL-1*β* and IFN-*γ* expression, and macrophage activation [12]. Similar findings are reported for patients with ALPS or DALD, who show increased levels of serum OPN and an increased risk of developing the diseases in subjects carrying the* OPN* haplotype B or haplotype C causing production of high levels of OPN. We proposed that high levels of OPN may contribute to the disease by inhibiting lymphocyte apoptosis, worsening the defect due to defective FAS function [14]. In particular, OPN directly inhibits activation-induced lymphocyte apoptosis and promotes secretion of TIMP1 [105] and IL-17 [106], which, in turn, inhibit both Fas-induced and activation-induced lymphocyte apoptosis.

Increased levels of OPN have been reported also in the sera and plasma of SLE patients [16, 84, 107], and their use has been suggested in monitoring SLE severity [107]. In line with these observations, a prospectic study suggested that high plasma levels of OPN may be predictors of poor outcome and are associated with the presence of autoAbs anti-ds-DNA and elevated IFN-*α* levels in the serum [108]. Moreover, high OPN levels in the serum and glomeruli are associated with renal damage, possibly mediated by the OPN's ability to support secretion of Th1 and Th17 proinflammatory cytokines and inflammatory cell migration and activation [107, 109, 110].

Recently a meta-analysis revealed that the OPN level was significantly higher in SLE patients and particularly in those with renal disease [25]. Moreover, it showed a trend of positive correlation between OPN levels and the systemic lupus erythematosus disease activity index (SLEDAI) [25]. The relationship between OPN and SLE has been confirmed by genetic studies showing correlations between* OPN* polymorphisms and development of SLE and suggesting that* OPN* may participate in a complex network of gene-gene and gene-environment interactions accounting for the SLE clinical heterogeneity. In line with this view,* OPN* polymorphisms have been correlated with specific clinical features of the disease, such as thrombocytopenia and hemolytic anemia, renal disease and opportunistic infections, lymphadenopathy, and high serum levels of IgE. Moreover, a correlation has been found with high serum levels of IFN-*α*, which is a key cytokine in SLE pathogenesis [16, 20, 22, 111, 112]. Some studies have detected a predominant influence of some polymorphisms on SLE development in males, who account for about 10% of SLE patients, which suggests that the OPN effect may be highlighted in the absence of strong SLE-promoting factors acting on females [113]. However, other reports have detected the same polymorphism as SLE-promoting factors in females and particularly in the young ones [16, 111], which may be ascribed to OPN's role in long bone remodeling during adolescence [114]. Other studies investigated* OPN* 9250C/T (rs1126616) polymorphism as SLE susceptibility variants, in association with OPN levels and clinical outcome. Authors found that the frequency of TT genotypes was higher in SLE patients with nephritis compared to controls, suggesting that the CT and TT genotypes could be risk factors for SLE [115]. Recently, Lee and Song conducted a meta-analysis on the role of OPN in SLE. They found that elevated OPN levels positively correlated with SLE activity and demonstrated a significant association between* OPN* 1239C/A, 9250C/T polymorphisms, and SLE susceptibility [25].

Interestingly, OPN in kidneys may also be secreted by senescent myofibroblasts and drive glomerular fibrosis [116]. OPN is required for myofibroblasts differentiation, and it regulates the molecules involved in tissue fibrosis. High levels of glomerular OPN are associated with macrophage accumulation and progressive renal injury in an antiglomerular basement membrane (GBM) glomerulonephritis (GN) model [116, 117]. OPN may modulate also angiotensin-II- (AII-) induced inflammation, oxidative stress, and fibrosis of the kidney [118]. Moreover, studies showed a localization of OPN at the origin of the fibrotic lesion in Bowman's capsule and the OPN deposition colocalized to the fibrotic lesion [116].

OPN also has an important function in vascular inflammation. It acts through modulation of the proliferation, migration, and accumulation of smooth muscle and endothelial cells [116, 119]. Under injury conditions, OPN plays a regulating role in arterial mineral deposition and in atherosclerotic lesions. OPN levels are high in human atherosclerotic lesions, and in lesions of ApoE^−/−^ mice, a model of atherosclerosis and aneurysm formation especially associated with macrophage and foam cells [120]. In vivo the function of OPN in atherosclerotic plaque formation has been proven by backcrossing ApoE^−/−^ mice with OPN^−/−^ mice. Double knockout mice highlighted the role of OPN in recruiting leukocytes, in macrophage apoptosis, and in reduction of AII-induced aortic aneurysm formation and MMP-2 and MMP-9 activity. These data support the idea that OPN and MMP have a role in regulation and vessel rupture [120].

### 2.2. Multiple Sclerosis

MS is a chronic inflammatory disease of the Central Nervous System (CNS). At onset, approximately 15% of patients display a primary progressive course (PP), whereas the others display a relapsing-remitting (RR) course, which mostly switches to a secondary progressive (SP) course within 10–15 years [121]. Analysis of the transcriptome has identified more than 50 genes highly overexpressed in MS lesions, and they included OPN [122]. High OPN levels have been reported in the serum, plasma, and cerebrospinal fluid (CFS) of MS patients and levels are higher in RR MS than in PP and SP MS, especially during the relapses. Moreover, OPN is expressed in reactive astrocytes and microglial cells in patients with RR-MS, particularly during the relapses [123–128]. The high OPN levels are positively correlated with the levels of IL-17 [129, 130].

Genetic analyses have associated variations of the* OPN* gene with MS [21, 131]. In this regard, we found that the variants of the* OPN* gene coding for a mRNA with increased stability (haplotype B and haplotype C) were associated with increased risk of MS, severe disease course, and rapid evolution of disability [132]. Furthermore, we detected a correlation between the 156G>GG SNP in the* OPN* promoter region and timing of disability progression and switch to the SP form [15, 132]. In Japanese patients, an* OPN* SNP (i.e., 9583A>G) has been associated with age of onset of disease [21]. However, other studies have not found associations between OPN variants and MS development or course [131].

Experimental autoimmune encephalomyelitis (EAE), the animal model of MS, is mediated by myelin specific autoreactive Th1 and Th17 cells. OPN deficient mice develop an attenuated form of EAE with a single relapse and lack of exacerbations or progression [79]. From the immunological point of view, they show a shift toward a Th2 cytokine profile, a reduction of proinflammatory cytokines, such as IFN-*γ*, and TNF-*α*, and increased numbers of apoptotic immune cells in the CNS lesions. Moreover, daily treatment with recombinant OPN worsened EAE in OPN^+/+^ mice and the effect was even more striking in OPN deficient mice. These results suggest that OPN influences the disease course not only by supporting expression of proinflammatory cytokines, but also by inhibiting apoptosis of autoreactive immune [133]. Treatment with IFN-*β* suppressed the production of IL-17 and OPN, through activation of signal transducer and activator of transcription (STAT)1 and suppression of STAT3 activity, and decreased spinal cord infiltration of cells secreting OPN and IL- 17 [130].

Proteomic studies performed on MS lesions have detected the expression of several molecules of the coagulation cascade and administration of coagulation inhibitors, such as hirudin, decreased disease severity, and suppressed production of Th1 and Th17 type cytokines [79, 133, 134]. These effects may involve OPN, since thrombin cleavage unmasks the OPN cryptic domain interacting with *α*4*β*1 integrin. This plays a key role in the recruitment of autoimmune T cells in MS lesions and is targeted by natalizumab, a humanized monoclonal antibody active in the treatment of RR MS [135]. Moreover, *α*4*β*1-OPN interaction prevents nuclear translocation of the transcription factor forkhead box O3A (FOXO3A), blocking transcription of proapoptotic genes such as Bim, Bcl-2 homologous antagonist killer (Bak), and Bcl-2-associated X protein (Bax), and promotes degradation of Ik-B with activation of NF-kB and upregulation of antiapoptotic genes and Th1 and Th17 cytokines [136].

A modulatory effect on OPN activity may also be exerted by OPN cleavage by MMPs whose levels are increased in several autoimmune diseases [45]. In line with this view, MMP-12-deficient mice develop more severe EAE than wild type mice, and this effect disappears in OPN/MMP-12 double-deficient mice [46, 47].

It has been reported that, after spinal cord injury, OPN is expressed by microglia and correlated with cell infiltration. OPN plays a major role in attracting inflammatory cells to the injury site [137] and, for a long time, most authors highlighted the role of OPN in exacerbating tissue damage after spinal cord injury. By contrast, Hashimoto et al. observed that the expression of Bcl-2, TNF-*α*, IL-1*β*, and IL-6 is downregulated in OPN knockout spinal cord after spinal cord injury that may result from a deficiency of OPN's proinflammatory activity [138]. Lower cytokine expression is accompanied by reduction of the number and activity of microglia/macrophages. Moreover, KO mice showed lower Basso Mouse Scale (BMS) scores than in wild type mice. These findings suggest that OPN is beneficial for recovery from spinal cord injury and plays a neuroprotective role during inflammatory response to spinal cord injury. This is also supported by research showing an upregulation of OPN, secreted by astrocytes and microglial cells, during the demyelination and remyelination, at the site of spinal cord injury [139]. A stroke model, too, in which focal cerebral ischemia is induced by photothrombosis, has shown a neuroprotective and regenerative function of OPN, especially when it is cleaved by thrombin [140].

### 2.3. Rheumatoid Arthritis

RA is a chronic, disabling autoimmune disease characterized by an inflammatory attack of the joint space, leading to the invasive growth of the synovial tissue and progressive destruction of the articular cartilage and bone [141]. RA patients have a shortened life span, and they suffer cardiovascular diseases caused by accelerated atherosclerosis which are a common cause of death in these patients. In the diseased joints, several cytokines are highly expressed, including OPN together with IL-1 and TNF-*α* [142].

The first evidence of a relationship between OPN and RA was provided in OPN^−/−^ mice that are protected from joint destruction in collagen-antibody-induced arthritis, an RA animal model (CAIA) [84]. High levels of OPN have been reported in the plasma and synovial fluid of RA patients [143] and have been associated with clinical severity indexes [144]. Furthermore, OPN mRNA is highly expressed in CD4^+^ synovial T cells and correlates with coexpression of selected OPN receptors, including *α*v and *β*1 integrin chains and CD44 [145]. Moreover, OPN plasma levels decrease after treatment with biologics or immunosuppressive drugs [145].

The effects of OPN on bone resorption are mainly ascribed to the interaction with CD44 and *α*v*β*3 integrin expressed by osteoclasts in the site of bone erosion [146]. However, other receptors may also be involved, since blocking *α*v*β*3 integrin by means of anti-*β*3 antibodies or other specific antagonists, such as SB273005 or cyclic RGD peptides, inhibits bone resorption only partly in animal models of RA [147].

Integrins *α*4 and *β*9 chains are expressed in arthritic joints and mAbs against the cryptic domain of OPN ameliorate collagen-induced arthritis by decreasing infiltration of inflammatory cells, proliferation of synovium, and development of bone erosions [148]. In line with a role of the cryptic domain, high levels of thrombin, OPN-N, and OPN-C have been detected in the synovial fluid of patients with RA. The levels of OPN-C and OPN-N correlate with the disease's severity, and the thrombin inhibitor hirudin ameliorates collagen-induced arthritis [149]. Moreover, the activity of OPN-N may be inhibited by thrombin-activatable carboxypeptidase B (CPB), which cleaves the C-terminal arginine from the cryptic site [150]. Interestingly, RA synoviocytes express a modified form of OPN, forming a complex with fibronectin and thus exposing the cryptic domain that promotes secretion of IL-6 in B cells [151].

In RA, OPN may play a crucial role by promoting differentiation of Th17 and Th1 cells, whose levels are elevated in rheumatoid synovium and correlate with several parameters of inflammation in RA patients [152]. Genetic studies did not detect univocal associations of* OPN* variants with RA. Some studies did not find any association between* OPN* polymorphisms and susceptibility to RA nor any correlation between OPN levels and* OPN* genetic polymorphisms [153, 154]. By contrast, an Italian study associated a SNP in the* OPN* promoter region (−156G>GG) with RA susceptibility [155]. These differences may be ascribed to the different influence of other concurrent genetic or environmental factors in the different populations of patients.

A recent prospective study, conducted in biologic-naïve patients with RA, pointed out that low OPN serum levels at baseline predict clinical remission one year after initiating tocilizumab treatment but not infliximab treatment [156].

OPN is also a crucial regulator involved in osteoarthritis (OA) progression [157]. OA is the most common form of arthritis, which mainly affects older people. OPN is highly expressed in the joints of OA patients, and its levels correlate with the severity of joint lesion and inflammatory status in the OA patients [158]. Abnormal mechanical load to chondrocytes alters the composition and metabolism of articular cartilage [159], which induces the release of OPN, and the enhanced level of OPN in cartilage leads to the induction of MMP-13 [160]. MMP-13 is thought to be involved in the degradation of cartilage matrix components of type II collagen and the release of proteoglycan from cartilage tissue that promotes the development of OA [161]. Elevated levels of OPN in cartilage also activate the transcription factor NF-*κ*B pathway involved in the production of many inflammatory factors, including chemokines and cytokines (e.g., IL-1, IL-6, IL-8, CXCL1, and CCL2) in cartilage, which leads to the spontaneous production of nitric oxide (NO), prostaglandin E2 (PGE2), IL-1*β*, IL-6, and IL-8. The overproduction of these cytokines and mediators exerts injurious effects on chondrocyte functions which lead to an imbalance of cartilage homeostasis resulting in progressive articular degeneration [162].

### 2.4. Other Autoimmune Diseases

#### 2.4.1. Type 1 Diabetes Mellitus (T1DM)

In T1DM, the autoimmune process is marked by the presence of antiglutamic acid decarboxylase (GAD), anti-islet cell, or anti-insulin antibodies, but the disease is mainly due to cell-mediated destruction of insulin-producing pancreatic *β*-cells [163]. This destruction is preceded by insulitis, a massive invasion of the islets by a mixed population of lymphocytes and macrophages. OPN may play a role in this inflammation since serum levels are increased in T1DM compared to controls and in diabetic patients correlated with high systolic and diastolic blood pressure, body mass index, low high density lipoprotein, diagnosis of retinopathy, and microalbuminuria [164, 165]. High OPN concentrations are associated with an unfavorable metabolic profile in these patients and are strong predictors of incipient diabetic nephropathy [164, 166]. SNPs analysis of the* OPN* gene showed that +1239C carriers displayed a significantly higher risk of T1DM than +1239A homozygotes [13]. Another SNP that can have a susceptibility role in T1DM development is located in the position 66. The G allele had significantly higher frequency in controls than T1DM patients. Interestingly, case-control comparison in males showed no significant association, whereas the association was confirmed in females [19]. Intriguingly, a screening of random peptide libraries with sera of young T1D patients detected an epitope of human OPN as an autoantigen expressed in the somatostatin cells of human islets [167].

#### 2.4.2. Sjögren's Syndrome (SS)

SS is an autoimmune disease characterized by lymphocyte infiltration of exocrine glands but can also involve the lungs, kidneys, and nervous system. Moreover, patients with SS are predisposed to develop non-Hodgkin's B cell lymphoma at a substantially higher rate than the general population. Recent studies suggested a role for OPN in SS pathogenesis [168, 169] since OPN levels are increased in the serum and salivary glands of patients with SS. Transgenic mice expressing OPN under the immunoglobulin enhancer/SV40 promoter spontaneously develop SS and display increased OPN levels both in the salivary glands and systemically. In tissues, OPN colocalizes with the inflammatory infiltration and is associated with reduced saliva production and increased autoantibody levels. These data have been confirmed in both the serum and submandibular salivary gland tissue using the well-characterized NOD/ShiLtJ mice, developing spontaneous SS disease in a highly predictable time frame [170]. Since OPN transgenic mice showed elevated OPN levels especially in B cell, B cell derived OPN has been speculated to play a role in SS development [90], even if a role may be played also by T cell derived OPN, and iOPN in TFH cells [73].

#### 2.4.3. Inflammatory Bowel Diseases (IBD)

IBDs are immune-mediated diseases typically resulting from abnormal mucosal T cell response to commensal bacteria in intestine and involve chronic intestinal inflammation, mucosal damage, and epithelial barrier dysfunction. Plasma concentration of OPN is elevated in patients with ulcerative colitis (UC) and correlate with clinical activity [81]. Moreover, Crohn's disease (CD) patients show elevated OPN expression in the terminal ileum and elevated plasma OPN levels correlate with disease activity [171–175]. In particular, in patients with active disease, the plasma OPN levels were increased compared with the quiescent disease and reduced after infliximab treatment. Genetic studies also found association of OPN haplotypes with CD susceptibility, and the combined effects of certain OPN variants may modulate IL-22 secretion [10]. However, preclinical studies showed that, in the acute phase of colitis, OPN-KO mice showed more extensive colonic ulcerations and mucosal destruction than wild type mice and the clinical phenotype was ameliorated by delivery of OPN. By contrast, in chronic dextran sulfate sodium- (DSS-) induced colitis, in which a Th1 response of the lamina propria infiltrates played a pivotal role, OPN-KO mice were protected from mucosal inflammation and produced less serum levels of IL-12 than wild type mice. Furthermore, neutralization of OPN was therapeutic in these mice. These data suggest a dual function of OPN in intestinal inflammation: during acute inflammation it activates innate immunity, reducing tissue damage and initiating mucosal repair; during chronic inflammation, it activates adaptive Th1 response, reducing inflammation [176].

### 2.5. Targeting OPN in Autoimmune Diseases

Patients with with RA or OA spontaneously produce anti-OPN autoAbs, and their serum levels are inversely correlated with markers of disease activity [177]. These data are in line with a large body of data showing that autoAbs against inflammatory cytokines can be detected in several inflammatory diseases and suggesting that they are a physiological mechanism to counteract the pathological effects of these cytokines [178]. In line with this model, induction of EAE promotes the production of anti-OPN autoAbs, and remission occurs when their titers peak. Furthermore, DNA vaccination with a plasmid encoding OPN before EAE induction boosts the production of these autoAbs and ameliorates the course of the disease [179]. In MS, we found increased levels of anti-OPN autoAbs in RR MS patients especially in the early phases of the disease and during the remission phase. Moreover, high levels of anti-OPN autoAbs at diagnosis correlate with relented development of disability in the RR MS patients treated with immunomodulating therapy.

These data suggest that the use of antagonists of OPN may be effective in the treatment of autoimmune diseases. In line with this possibility, injection of anti-OPN antibodies ameliorates the disease in primate and mouse RA [84]. These experiments showed that this cryptic epitope is involved in leukocytes migration, cell adhesion, cytokine production, and progression of arthritis [180]. Moreover, in a rat model of antiglomerular basement membrane glomerulonephritis, induced by immunizing mice with the nephritogenic T cell epitope pCol(28–40) derived from *α*3 chain of type IV collagen, treatment with neutralizing antibodies to OPN inhibited development of glomerular fibrosis [117].

These results obtained in animal models have prompted a recent study on safety, tolerability, pharmacokinetics, pharmacodynamic, and efficacy of a monoclonal antibody against OPN in patients with RA [180]. Patients with RA were divided into two random groups, that is, treated with a placebo or a humanized IgG1 monoclonal antibody (ASK8007) directed to the cryptic site of OPN and inhibiting both RGD- and *α*9*β*1 integrin-dependent cell binding to human OPN [180, 181]. Overall ASK8007 administration appeared safe and well tolerated up to a highest dose (20 mg/kg), but it did not induce any improvement in joint inflammation and destruction. This can be the consequence of the clinical trial design (early time of evaluation, low power of the study, and aggressive disease compared to the mild preclinical one), low affinity of the mAb for the human OPN, or the methodology used targeting OPN but not iOPN. To overcome this problem, silencing OPN expression might be a good approach since mice with collagen-induced arthritis ameliorated the inflammatory response and bone destruction (articular swelling and cartilage erosion) in the ankle joint upon inhibition of OPN expression, by mean of lentiviral OPN short hairpin RNA [182]. Moreover, preclinical data on experimental autoimmune uveitis, in which targeting of OPN has been obtained by mean of a small interfering RNA (siRNA) [183], are encouraging.

Results showed that plasma levels of OPN and the clinical and histopathological scores of disease were lower in the siRNA-treated group than that in the control group.

## 3. Conclusions

OPN is multifaceted protein exerting several roles in inflammation, adaptive immunity, tissue repair, bone formation, and cell signaling. These heterogeneous activities may be ascribed to the multiple variants of OPN including those due to transcriptional, posttranscriptional, and posttranslational modifications. These variants may be variably involved in the pathogenesis of different autoimmune diseases and clarification of the role of each variants is crucial to tailor appropriate therapeutic approaches targeting this complex molecule.

## Figures and Tables

**Table 1:** *OPN* gene polymorphisms associated with autoimmune diseases.

SNP	rs	Located	Autoimmune disease	References
−156 G>GG	Rs7687316	Promoter	MS, T1D, SLE	[15, 16, 19]
−66 T>G	Rs28357094	Promoter	T1D, CD, SLE	[10, 19, 20]
+282 T>C (8090TH)^$^	Rs4754	Exon VI	MS, SLE, ALPS/DALD, RA, CD	[10, 14, 21]
+750 C>T (+707; 9250TH)^$^	Rs1126616	Exon VII	SLE^#^, PBC, MS, CD, ALPS/DALD	[10, 14, 21–23, 25]
+1083 A>G (9583RD)^$^	Rs1126772	3′UTR	BD, SLE^*∗*^, MS, ALPS/DALD, CD	[10, 13, 14, 16, 21, 24, 25]
+1239 A>C	Rs9138	3′UTR	CD, TDM1, MS, SLE, ALPS/DALD	[10, 13–16]

^#*∗*^A recent meta-analysis found an association only in Asian but not in European SLE patients (#) and no association with SLE patients (*∗*) [25].

^$^SNPs may have different numbering depending on the transcript analysed and the assembly used.
